# Changes in autonomic nervous system activity, body weight, and percentage fat mass in the first year postpartum and factors regulating the return to pre-pregnancy weight

**DOI:** 10.1186/s40101-016-0115-5

**Published:** 2016-10-27

**Authors:** Mie Izumi, Emiko Manabe, Sayo Uematsu, Ayako Watanabe, Toshio Moritani

**Affiliations:** 1 Department of Nursing, Doshisha Women’s College of Liberal Arts, Koudo Kyotanabe City, Kyoto, Japan 610-0395; 2 Graduate School of Psychological Science, Hiroshima International University, Kurosegakuendai Higashihiroshima City, Hiroshima, Japan; 3 Graduate School of Human and Environmental Studies, Kyoto Sangyo University, Motoyama Kamigamo Kita-ku Kyoto City, Kyoto, Japan

**Keywords:** Autonomic nervous system, Body mass index, Postpartum, Period/ME

## Abstract

**Background:**

Many women become obese during pregnancy and the postpartum period. Weight gain and obesity in the general population are often attributed to abnormalities of autonomic nervous system (ANS) activity. The aim of this study was to clarify change in ANS activity, body weight, percentage fat mass (%FM), and body mass index (BMI) and the factors regulating the return to the pre-pregnancy weight in the first year postpartum.

**Methods:**

This study was conducted from 2012 to 2016 at the University Hospital of the Kyoto Prefectural University of Medicine and a nearby obstetrics and gynecology clinic in Japan. Body weight and %FM were measured in 51 women using a dual-frequency body composition measuring device. Heart rate variability and R–R spectral transformation were used as indicators of ANS activity. All parameters were calculated at three postpartum time points. Repeated measure analysis of variance was used for comparisons between measurement times. A multivariable Cox proportional hazards model was conducted to determine factors associated with the return to pre-pregnancy weight.

**Results:**

Mean body weight, %FM, and BMI decreased significantly over time after delivery (*P* < 0.001, *P* < 0.001, *P* < 0.001). However, ANS activity did not differ among subjects in the three time points. 25.5 % of subjects had still not returned to their pre-pregnancy body weight by 150–270 days postpartum, and 19.6 % had not by 270–360 days postpartum. Normal-weight obesity (NWO; BMI of 18.5–25 kg/m^2^ and %FM of ≥30 %) was observed in several subjects at each measurement. The results of analysis using a multivariable Cox proportional hazards model suggest that ANS activity had no significant correlation with the return to pre-pregnancy weight.

**Conclusions:**

The management of body weight and %FM after delivery is considered important. These findings suggest that ANS activity is not associated with the return to pre-pregnancy weight, albeit that sample size was small.

## Background

Obesity (body mass index (BMI) ≥ 30) is defined as a condition of abnormal or excessive fat accumulation that may impair health [1]. An elevated BMI is a major risk factor for cardiovascular diseases (mainly heart disease and stroke), diabetes, and some cancers. Although once considered problems only affecting high-income countries, the prevalence of overweight and obesity is now dramatically increasing in low- and middle-income countries as well, particularly in urban areas. In 2014, more than 1.9 billion adults (18 years and older) were overweight, and of these, over 600 million were obese [1]. Overall, approximately 13 % of the world’s adult population (11 % of men and 15 % of women) was obese in 2014 [1].

Female obesity often begins during pregnancy or soon after childbirth [2]. Promoting health management tailored to the stage of life may ensure that changes in body weight and physique caused by pregnancy and childbirth are not overlooked. With many women choosing to marry and have children later in life, a trend towards older women becoming pregnant is increasing in Japan. Women who become pregnant at an older age often find it difficult to return to their pre-pregnancy body weight [3]. Since weight gain during pregnancy is associated with a variety of perinatal risks [4–6], the United States Institute of Medicine (IOM) has published guidelines that have been revised by the Japanese Ministry of Health, Labor and Welfare on how much weight a woman should gain during pregnancy, highlighting the importance of intervention during pregnancy to prevent postpartum weight retention [7, 8].

Women with normal-weight obesity (NWO; normal BMI with an elevated percentage fat mass (%FM) ≥ 30 %) are said to be at increased risk of metabolic disorders and cardiovascular disease [9], underscoring the importance of weight management using %FM as a useful marker in addition to BMI. However, few investigations have evaluated the changes in the %FM of postpartum women.

The MONA LISA hypothesis [10] proposed in 1991 purports that autonomic nervous system (ANS) activity and the energy metabolism regulatory functions of the sympathetic nervous system have a major influence on obesity and fat metabolism, highlighting the possibility of ANS activity being a cause of weight gain and obesity. ANS activity is also thought to be involved in the course of postpartum body weight and %FM, but details regarding these relationships remain unclear.

The aim of this study is to clarify the change of ANS activity, body weight, percentage fat mass (%FM), and body mass index (BMI) and the factors regulated to the return to the pre-pregnancy weight in the first year postpartum.

## Methods

This study was conducted from 2012 through 2016. Subjects were recruited from among mothers who gave birth at the University Hospital, Kyoto Prefectural University of Medicine (Kyoto, Japan), and a nearby obstetrics and gynecology department clinic. As shown in Table 1, significant differences in socioeconomic and demographic characteristics were not found between the two health care center subgroups. Further, all subjects neither smoked nor drank from before pregnancy, and all subjects were married. We displayed a written summary (including the date and time, location, and the methods) of the study at the facilities. We studied 51 mothers with no underlying diseases who agreed to participate in three surveys from 1 to 12 months after delivery (first survey: 30–150 days, second survey: 150–270 days, third survey: 270–360 days). The nature and purpose of the study were explained to all participants, who gave written informed consent to participate. The study protocol was approved by the Ethics Review Board of Kyoto Prefectural University of Medicine.

**Table 1:** Socioeconomic status and demographic characteristics of the participants

	ALL (*n* = 51)	University Hospital, Kyoto Prefectural University of Medicine (*n* = 17)	Obstetrics and gynecology department clinic (*n* = 34)	*P* values
Age	33.7 ± 0.6	34.1 ± 1.0	33.5 ± 0.7	0.554
Employment status^ab^				
Employed	31(60.8)	8(15.7)	23(45.1)	0.156
Unemployed	20(39.2)	9(17.6)	11(21.6)	
Family structure^ab^				
Nuclear family	50(98.0)	17(33.3)	33(64.7)	1.000
Extended family	1(2.0)	0	1(2.0)	
Period of pregnancy (week)	39.2 ± 0.2	39.4 ± 0.3	39.1 ± 0.2	0.633
Pre-pregnancy BMI (kg/m2)	20.1 ± 0.3	20.0 ± 0.6	20.2 ± 0.4	0.849
Pre-pregnancy BMI group^ab^				
BMI of <18.5	14(27.5)	6(11.8)	8(15.7)	0.555
BMI of 18.5-25.0	35(68.5)	10(19.6)	25(48.9)	
BMI of > 25.0	2(4.0)	1(2.0)	1(2.0)	

Mean ± standard error or ^a^N (%); Mann-Whitney-U test or ^b^χ^2^ test

### Questionnaire survey

Subjects completed a standardized questionnaire which included age, occupation, family structure, marital status, pre-pregnancy and postnatal exercise habits, medical history (mother and baby), parity, day of delivery, expected delivery date, method of childbirth, breast feeding, height, pre-body weight, and gestational weight gain.

### Measurement of body weight and fat mass

Body weight and %FM were determined using a bioelectrical impedance analyzer (BIA) (DC-320, Tanita Corp., Tokyo, Japan) [11]. Subjects wore indoor clothing with no shoes. Half a kilogram (0.5 kg) was deducted from the weight of each participant to account for the weight of clothing. The %FM was calculated as fat mass/weight × 100, and BMI was calculated as the weight (kg)/height (m)^2^. The study defined +1 kg difference from the pre-pregnancy weight as a return to the pre-pregnancy weight. Subjects who returned to the pre-pregnancy weight within a year of delivery were categorized as the weight loss group, and those who did not were categorized as the no weight loss group.

NWO was defined as a BMI of 18.5–25 kg/m^2^ and a %FM of ≥30 %. We defined “pre-NWO” as a BMI of 18.5–25 kg/m^2^ and a %FM of 25–30 %.

### Measurement of ANS activity

All examinations were performed in the Kyoto Prefectural University of Medicine or the nearby obstetrics and gynecology department clinic. All data acquisition was performed in our investigation room, which was kept quiet and comfortable, at a temperature of approximately 25–28 °C. For the assessment of ANS activity, after at least 20 min of rest, and confirmation that participants did not have tachycardia or hypertension, the investigation was started [12]. Subjects were fitted with a photoplethysmography (PPG) monitor (Biocom Technologies, Inc., Washington DC, USA) on the earlobe, which acquired electrocardiographic (ECG) data for 5 min. The ECG was assessed for heart rate variability (HRV) with an analyzer (Heart Rhythm Scanner; HRV Analysis System; Biocom Technologies, Inc.) equipped with software that performs short-term HRV analysis algorithms. This system was based on the recommendations of the European Society of Cardiology and the North American Society of Pacing and Electrophysiology [13]. The reliability of these instruments has been confirmed by comparing the results of measurements using PPG with those of 12-lead ECG [14, 15]; this technique has also been used by Suetake et al. [16]. Subjects were asked to listen to a metronome set at 15 clicks/min and synchronize their breathing rate accordingly [12].

### R–R spectral analysis

We assessed resting ANS activity by noninvasively analyzing HRV using power spectral analysis of the ECG. Cardiac rhythm is modulated by the sympathetic and parasympathetic components of the ANS, which exert antagonistic effects. The R–R interval on ECG, the inter-beat interval of heart rate, is determined by the net effect of inputs from the sympathetic and parasympathetic systems [13]. This interval constantly fluctuates, and the extent of fluctuation constitutes HRV, which is a surrogate for ANS activity [13, 17]. Spectral transformation of this R–R interval variability generates a high-frequency band (HF; approximately 0.15–0.4 Hz), a low-frequency band (LF; approximately 0.04–0.15 Hz), and a very-low-frequency band (VLF; approximately 0.003–0.04 Hz). In addition, the sympathetic nervous system (SNS) and parasympathetic nervous system (PNS) activities were also calculated as ratio of (VLF + LF)/HF and HF/TOTAL, respectively, according to the previous studies [18].

### Statistical analyses

All statistical analyses were performed using a commercial software package (SPSS 24.0 J for Windows; SPSS Inc., Tokyo, Japan). Since variation was large, the TP at the first survey was considered to be 100, and TP, LF, HF, VLF, LF/HF, SNS, and PNS activities were normalized by this TP at first survey value. Repeated measure analysis of variance was used for comparisons between measurement times. A multivariable Cox proportional hazards model was conducted to determine factors associated with the return to pre-pregnancy weight in the first year postpartum. Return or not to the pre-pregnant weight was used as the objective variable, while SNS activity, PNS activity, pre-pregnancy exercise habits, parity, pre-pregnancy BMI, and age were used as explanatory variables. The category division of SNS activity, PNS activity, BMI of pre-pregnancy, and age were carried out based on average value. All *P* values less than 0.05 were considered statistically significant. Data are expressed as mean ± standard error.

## Results

Table 2 shows the demographic characteristics of the participants, in total and by parity. Significant differences were observed only for age (*P* < 0.05). All subjects had single pregnancies and vaginal delivery, and all their children were normal.

**Table 2:** Demographic characteristics of the participants

	ALL (*n* = 51)	Primipara (*n* = 35)	Multipara (*n* = 16)	*P value*
Age	33.7 ± 0.6	32.8 ± 0.7	35.6 ± 0.935.6 ± 0.9	0.029
Height (cm)	159.0 ± 0.7	159.2 ± 0.9	158.7 ± 1.2	0.831
Pre-pregnancy body weight (kg)	50.9 ± 1.0	49.9 ± 1.1	53.3 ± 2.0	0.102
Pre-pregnancy BMI (kg/m^2^)	20.1 ± 0.3	19.6 ± 0.3	21.2 ± 0.8	0.090
Pre-pregnancy BMI group^ab^				
BMI of <18.5	14(27.5)	11(21.6)	3(5.9)	0.081
BMI of 18.5-25.0	35(68.6)	24(47.0)	11(21.6)	
BMI of > 25.0	2(3.9)	0	2(3.9)	
Gestational weight gain (kg)	9.5 ± 0.5	9.5 ± 0.6	9.7 ± 1.1	0.959
Period of pregnancy (week)	39.2 ± 0.2	39.5 ± 0.2	38.5 ± 0.5	0.069
Exercise habits in pre-pregnancy^ab^				
have	9(17.6)	8(15.7)	1(2.0)	0.242
no	42(82.4)	27(52.9)	15(29.4)	
Breast feeding				
have	43(84.3)	30(58.8)	13(25.5)	0.694
no	8(15.7)	5(9.8)	3(5.9)	

Mean ± standard error or ^a^N (%); Mann-Whitney-U test or ^b^χ^2^ test weight loss group, no weight loss group

### Body weight, %FM, and BMI

Body weight, %FM, and BMI at each measurement time are also shown in Table 3. Body weight, %FM, and BMI decreased significantly over time after delivery (*P* < 0.001, *P* < 0.001, *P* < 0.001). However, 13 (25.5 %) subjects had not returned to their pre-pregnancy body weight by the second measurement, and 10 (19.6 %) had still not returned to their pre-pregnancy body weight by the third measurement. Of the 35 subjects who had average pre-pregnancy BMI, 8 (22.9 %) had not returned to their pre-pregnancy body weight by the second measurement and 7 (20.0 %) had not returned by the third measurement.

**Table 3:** Body weight, percentage fat mass, body mass index, and normalized autonomic nervous system activity at three time points (*n* = 51)

	30–150 days	150–270 days	270–360 days	*P values*
Body weight (kg)	51.5 ± 1.0	50.4 ± 1.0	49.9 ± 1.0	<.001
Percentage fat mass (%)	27.4 ± 0.8	26.1 ± 0.8	25.3 ± 0.8	<.001
Body mass index	20.3 ± 0.3	19.9 ± 0.3	19.7 ± 0.3	<.001
Body weight reduction (kg)	8.8 ± 4.2	9.9 ± 4.4	10.4 ± 5.0	<.001
TP	100.0	122.5 ± 14.9	133.9 ± 18.6	0.216
LF	27.6 ± 1.6	39.9 ± 5.9	47.4 ± 8.3	0.081
HF	39.5 ± 2.5	45.4 ± 6.0	48.5 ± 7.1	0.473
VLF	32.8 ± 2.4	37.2 ± 4.5	37.9 ± 4.6	0.541
SNS activity	2.8 ± 0.6	2.1 ± 0.2	2.0 ± 0.2	0.662
PNS activity	0.4 ± 0.02	0.4 ± 0.02	0.4 ± 0.02	0.662
Exercise habits^a^				
have	4(7.8)	4(7.8)	4(7.8)	-
no	47(92.2)	47(92.2)	47(92.2)	

Mean ± standard error or ^a^N (%); repeated measures analysis of variance

*TP* total power, *LF* low frequency, *HF* high frequency, *VLF* very low frequency, *SNS* sympathetic nervous system, *PNS* parasympathetic nervous system

TP at 30-150 days was considered to be 100, and TP, LF, HF, VLF, SNS activity, and PNS activity were normalized by this value

Of the 51 subjects, NWO was observed in 12 subjects (23.5 %) at the first measurement, in 9 (17.6 %) at the second, and in 9 (17.6 %) at the third, and pre-NWO was observed in 20 (39.2 %) at the first measurement, in 16 (31.4 %) at the second, and in 15 (29.4 %) at the third. In addition, overweight (BMI ≥ 25 kg/m^2^) was observed in 3 (5.9 %) each at the first and second measurements, and in 1 (2.0 %) at the third measurement.

### ANS activity

Table 3 shows TP, LF, HF, VLF, LF/HF, SNS, and PNS activities normalized by the TP value at the first measurement point. No significant differences in activity were noted among the three time points.

### Comparison between the weight loss group and no weight loss groups

Average age was 33.9 (0.7) years in the weight loss group (*n* = 41) and 32.8 (0.8) years in the no weight loss group (*n* = 10). Among other variables, pre-pregnancy BMI was 20.3 (0.4) kg/m^2^ and 19.3 (0.5) kg/m^2^; the period of pregnancy was 39.3 (0.2) weeks and 39.0 (0.5) weeks; the number of primiparas was 28 (54.9 %) and 7 (13.7 %); the number of multiparas was 13 (25.5 %) and 3 (5.9 %); and the number of those who had exercise habits before pregnancy was 8 (15.7 %) and 1 (2.0 %), respectively. The results of ANS activity are indicated to Table 4. No significant differences were seen among the two groups for any item.

**Table 4:** Comparison between weight loss group and no weight loss group

	30–150 days		*P* values	150–270 days		*P* values	270–360 days		*P* values
	weight loss group	no weight loss group		weight loss group	no weight loss group		weight loss group	no weight loss group	
SNS activity	2.9 ± 0.8	2.1 ± 0.4	0.406	2.1 ± 0.3	1.9 ± 0.2	0.618	2.0 ± 0.2	2.0 ± 0.5	0.776
PNS activity	0.4 ± 0.03	0.4 ± 0.04	0.393	0.4 ± 0.02	0.4 ± 0.03	0.618	0.4 ± 0.02	0.4 ± 0.05	0.776

Mean ± standard error; Mann-Whitney-U test

*SNS* sympathetic nervous system, *PNS* parasympathetic nervous system

TP at 30-150 days was considered to be 100, and SNS activity, PNS activity were normalized by this value

### Factors related to the return to pre-pregnancy weight

To identify factors that affected the return to pre-pregnancy weight within a year of delivery, an analysis using a multivariable Cox proportional hazards model (forced entry method) was performed. All confidence intervals straddled 1, and the results showed no significant differences regardless of variable (Table 5).

**Table 5:** Analysis of factors affecting the return to pre-pregnancy weight using a multivariable Cox proportional hazards model

	Factor	Category advantage/disadvantage	Partial regression coefficient	*P* value	Hazard ratio	95 % CI
SNS activity^a^	First survey: 30–150 days after delivery	≦2.80/>2.81	−1.045	0.064	0.352	0.116 to 1.061
	Second survey: 150–270 days after delivery	≦2.10/>2.11	0.103	0.807	1.108	0.487 to 2.524
	Third survey: 270–360 days after delivery	≦2.00/>2.01	−0.987	0.069	0.373	0.129 to 1.079
PNS activity^a^	First survey: 30–150 days after delivery	≦0.4/>0.41	−0.903	0.071	0.405	0.152 to 1.080
	Second survey: 150–270 days after delivery	≦0.4/>0.41	−0.675	0.168	0.509	0.195 to 1.329
	Third survey: 270–360 days after delivery	≦0.4/>0.41	−0.464	0.374	0.629	0.226 to 1.750
Exercise habits in pre-pregnancy		±	0.219	0.623	1.244	0.520 to 2.975
Parity		Primipara/multipara	0.437	0.289	1.548	0.691 to 3.467
BMI in pre-pregnancy		≦24/≧25	−1.246	0.164	0.288	0.050 to 1.666
Age		≦34/≧35	−0.381	0.302	0.683	0.332 to 1.408

*95 % CI* 95 % confidence interval

^a^TP at 30–150 days was considered to be 100, and SNS and PNS activities were normalized by this value

## Discussion

The weight of mothers gradually approached the pre-pregnancy weight over 6 months after giving birth [19]. However, approximately 25 % of participants had not returned to their pre-pregnancy body weight by 150–270 days after delivery, and approximately 20 % had still not returned to their pre-pregnancy body weight by 270–360 days after delivery. In addition, sizeable percentages of subjects, depending on the measurement time, were found to have NWO or pre-NWO. High body weight—or more specifically, a high BMI—is associated with metabolic syndrome and elevated rates of morbidity and mortality [1, 20]. NWO is also associated with metabolic syndrome and cardiovascular diseases [21]. The prevalence of NWO is higher in women than in men [21], and BMI and %FM tend to increase with age [22]. In Japanese women in particular, the percentage of women thought to be at risk for metabolic syndrome increases with age, with frequencies of 1.4 % in the third decade, 6.1 % in the fourth decade, 8.0 % in the fifth decade, and 15.1 % in the sixth decade [23]. The average delivery age of 31.5 years among Japanese women [24] may explain the sharp increase between the third and fourth decades, as pregnancy and childbirth have been hypothesized to trigger metabolic syndrome. The age of menopause among Japanese woman is an average of 50 years [25], and the increase in the sixth decade may be attributable to menopause-related weight gain [26]. Thus, addressing %FM is seen as increasingly important, in addition to managing body weight postpartum and later in life.

ANS activity is involved in energy expenditure and appetite as well as in adjustment of body weight. Reductions in ANS activity, particularly decreases in sympathetic nervous activity, can lead to overweight [27]. It is therefore considered that body weight is affected by age, ANS activity, and exercise habits. Because the multiparas were older than the primiparas, parity is also considered to affect body weight. However, these factors had no effect on the return to pre-pregnancy weight for the following reasons: (1) there were no significant differences in age or ANS activity between those who returned to their pre-pregnancy weight within a year of delivery and those who did not; (2) only a small number of subjects were obese; (3) many subjects were likely to have had normal ANS activity; and (4) only a small number of subjects who had had exercise habits before pregnancy maintained their exercise habits after delivery.

## Conclusions

No marked postpartum changes were observed in ANS activity as body weight, %FM, and BMI decreased over a period of months following delivery. However, approximately 20 % had still not returned to their pre-pregnancy body weight by 270–360 days after delivery. In addition, NWO and pre-NWO were observed in some subjects at different measurement points, suggesting that managing weight and fat mass are both important for preventing postpartum obesity. Also, ANS activity was not correlated with the return to pre-pregnancy weight. These findings warrant confirmation in a larger population.

### Limitations and future directions

The low number of subjects and low percentage of obese subjects may limit the generalization of these results. In the future, we intend to examine a larger population of subjects to clarify postpartum weight changes in obese subjects and to examine the relationship or otherwise between changes in weight and ANS activity. In this study, ANS activity at rest was used for analysis. We will further investigate ANS activity using the Scholling test.
